# Putting Youth on the Map: A Pilot Instrument for Assessing Youth Well-Being

**DOI:** 10.1007/s12187-012-9170-6

**Published:** 2012-10-12

**Authors:** Nancy Erbstein, Cassie Hartzog, Estella M. Geraghty

**Affiliations:** 1 Department of Human Ecology, University of California, Davis, One Shields Avenue, Davis, CA 95616 USA; 2 Department of Sociology, University of California, Davis, One Shields Avenue, Davis, CA 95616 USA; 3 Department of Internal Medicine, Division of General Medicine, University of California, Davis, 4150 V Street, PSSB 2400, Sacramento, CA 95817 USA

**Keywords:** Adolescent well-being, Child well-being, Index, Spatial analysis, Metropolitan regions

## Abstract

Extant measures of adolescent well-being in the United States typically focus on negative indicators of youth outcomes. Indices comprised of such measures paint bleak views of youth and orient action toward the prevention of problems over the promotion of protective factors. Their tendency to focus analyses at a state or county geographic scale produces limited information about localized outcome patterns that could inform policymakers, practitioners and advocacy networks. We discuss the construction of a new geo-referenced index of youth well-being based on positive indicators of youth development. In demonstrating the index for the greater Sacramento, California region of the United States, we find that overall youth well-being falls far short of an optimal outcome, and geographic disparities in well-being appear to exist across school districts at all levels of our analysis. Despite its limitations, the sub-county geographic scale of this index provides needed data to facilitate local and regional interventions.

## Introduction

Over the past 20 years data on the status of children and youth have become increasingly available to policy-makers, practitioners, and advocacy networks in the United States. Existing resources from the United States Census Bureau and public agencies are now available online, and websites have been established to compile data from multiple sources—for example Kids Count (http://www.aecf.org/MajorInitiatives/KIDSCOUNT.aspx) and Child Trends Data Bank (http://www.childtrendsdatabank.org/) operate at the national level, and there are statewide and regional sites such as Kids Data (http://www.kidsdata.org/). With the availability of multiple, diverse data points, scholars have constructed indices that look across multiple domains to assess overall levels of youth vulnerability and well-being. For example, the Child Well-Being Index (CWI) is comprised of 28 items that are updated annually (Land et al. 2001; Land 2006); the Annie E. Casey Foundation employs a ten-item index to track child well-being across states and over time (O’Hare and Bramstedt 2003) and offers indicator data for counties and, in some cases, metropolitan statistical areas through a system of state partners.

The following paper builds upon these and other efforts through a geo-referenced index of youth well-being produced for the metropolitan region surrounding the state capital of California, Sacramento—referenced here as “California’s Capital Region.” The region covers 9,046 square miles, just slightly smaller than the state of New Hampshire, yet has more than double the population size at 2.87 million residents. This index was produced through the Healthy Youth/Healthy Regions initiative, a collaborative partnership between the UC Davis Center for Regional Change, the Sierra Health Foundation, and The California Endowment to document the connections between improvements in youth well-being and regional prosperity in the nine-county Sacramento Capital Region.^1^ The index extends existing work in four important ways: by employing positive indicators of youth development, by using both subjective and objective measures, by providing analyses of relatively small geographic units (school districts), and by presenting analyses of multiple measures in an accessible manner.

## Child and Youth Well-Being Index Development Challenges

Existing indices of child and youth well-being have evoked multiple critiques of their validity and utility. Conceptually, several scholars question whether there is consensus on the definition of “child well-being,” given the variety of approaches to framing and measuring it (Pollard and Lee 2003). Morrow and Mayall (2010) point out that the definition of child well-being is historically and culturally contingent, and has been variously defined as an aspect of health, happiness or “becoming.” These authors and others argue that youth, as actors and knowers, ought to be involved in the discourse, and that their exclusion is likely to lead to adult-centric measures of well-being (Fattore et al. 2007).

In recent years, several authors have also questioned the widespread use of negative indicators in child well-being indices. Measured this way, such indices do not represent well-being, but its opposite (Moore and Halle 2001). A focus on negative indicators skews our collective view of well-being, which is more than just the absence of negatives (Ben-Arieh et al. 2001). In addition, an exclusively negative orientation limits monitoring of positive assets and protective factors. For example, in the United States youth themselves cite feeling competent, valued, safe, and secure as important to their sense of well-being (Fattore et al. 2007), a point corroborated by youth development researchers (Lippman et al. 2009), yet related measures are rarely captured in indices of child well-being.

Several limitations undermine the utility of indices as tools to inform programming, policy development, and advocacy, particularly at a local and regional scale. Available resources typically employ data that are representative at a county or larger geography, masking more local patterns that might inform action. Data are rarely analyzed or presented in ways that facilitate understanding of geographic and temporal patterns. In fact, indices often include measures that are not readily available (i.e. relying upon specialized surveys), are infrequently updated, or include transformations on variables that preclude comparison with an optimal outcome and impair trend analyses over time (e.g. the use of z-scores based on a mean that shifts from time one to time two).

Despite these shortcomings, well-being indices can enhance our capacity to assess the conditions of youth, document disparities, and develop effective interventions (Ben-Arieh 2008). Addressing these challenges is an important next step in supporting evidence-based planning and advocacy at the local and metropolitan-regional scales, as well as state and national activity that accounts for more localized patterns. In recent years, researchers have been making strides in the development and analysis of regional well-being indices. For example, trends in child well-being and geographic disparities in well-being have been documented in the San Francisco, California region (Lee et al. 2009), in North Carolina counties (Hur and Testerman 2010), in health service delivery areas in British Columbia, Canada (Martin et al. 2011), neighborhoods in England (Bradshaw et al. 2009), and in municipalities in Greenland (Niclasen and Kohler 2009). These studies find significant regional variation in well-being and also document rural-urban disparities in well-being. Though like many of their predecessors, the majority of the indices used in these studies are comprised primarily of indicators of ill-being rather than well-being, with the exception of Martin et al. (2011). We advance the state-of-the-art by developing an index based on positive assessments of well-being that includes both subjective (based on youth self-reports) and objective indicators (from administrative data) measured at a local scale with careful consideration of future comparability and trend analysis.

## Methods

For the purpose of this effort, “youth well-being” refers to the personal, familial, and social conditions that enable adolescents to function well in multiple contexts (Lippman et al. 2009; Pollard and Lee 2003). While approaches to measuring the construct of well-being vary, there is general agreement that certain domains are essential (Ben-Arieh et al. 2001; Eccles and Gootman 2002; Moore et al. 2007; Pollard and Lee 2003). The domains encompass psychological, social, physical, cognitive, and economic aspects of well-being, and with the exception of the psychological and social domains, are well-represented in contemporary indices (Bradshaw et al. 2007, 2009; Hur and Testerman 2012; Land et al. 2001; Lee et al. 2009; Martin et al. 2011; Moore et al. 2008; O’Hare and Bramstedt 2003).

Drawing inspiration from these earlier efforts,^2^ we focus on four domains of well-being: health, education, social relationships, and community context. The health domain encompasses physical health, avoidance of risk behaviors, such as abstinence from alcohol and drug use, and physical and emotional safety/freedom from worry. The education domain refers to academic skills and educational achievement. The social relationships domain encompasses relationships with important others, recognizing that positive adolescent adaptation and development depends in part on the presence of supportive parents/caretakers, caring adults, close friends, and supportive environments (Bronfenbrenner and Morris 1998; Moore et al. 2008; Theokas and Lerner 2006). The community context domain examines environmental factors such as economic resources children experience in their households and communities, and participation in their communities.

Index development entailed four key steps. These include: (1) selection of appropriate data sources, (2) selection of indicators and measures, (3) index construction and data analysis, and (4) production of the maps.

### Data Sources and Their Limitations

Our project objectives implied several parameters for selecting data sources, including accessibility, regular data collection, inclusion of positive measures, representativeness at the most local scale possible, and shared geographic scale across data sets. As we assessed options in the context of these criteria and our research-based domain areas, three key sources emerged that enabled analysis at a shared sub-county scale—the school district: the U.S. Census Bureau, the California Department of Education (CDE), and the California Healthy Kids Survey (CHKS). Each proved to be an important resource but also presented some limitations.

The U.S. Census Bureau provides a rich source of data on youth and family conditions. Limitations of these data include potential under-representation of undocumented immigrant populations (a group that has been critical to this region’s economy) (Hoefer et al. 2007), as well as increased potential for error in population estimates for hard-to-count, low-density rural areas (U.S. Census Bureau 2009).

The CDE makes available a wealth of data on the academic progress of students in California, including standardized test results and high school completion and dropout rates, as well as course selections and physical fitness test results. Data are available at the school, district, county and state level. But importantly, while administration of standardized academic tests is fairly uniform across schools, physical fitness tests are administered by local teachers who receive limited instruction, introducing potential variability in the interpretation of these tests. In the past school data have suffered from an inability to track high-mobility students, undermining the accuracy of school and district graduation and dropout rates. This caveat applies to the CDE data presented here, which is from the 2006–07 school year and reflects a snapshot of student enrollment as reported by each school. In the future, a new statewide student tracking system will enable use of more accurate graduation and dropout data.

Though objective measures such as household income, physical fitness test scores and high school graduation rates are indicative of youth well-being, researchers increasingly also recognize the importance of self-reports regarding quality of life (Fattore et al. 2007; Lippman et al. 2009; Morrow and Mayall 2010). When asked to define well-being, youth commonly cite subjective and relational factors such as being happy, having positive friendships, feeling safe in their environment, and being able to act freely and make decisions for themselves in the context of stable and secure relationships with caring adults (Gilman et al. 2000). A growing body of literature supports the link between satisfaction with quality of life and multiple adaptive outcomes and mental states (Diener and Seligman 2002; Gilman and Huebner 2006). Life satisfaction is an important indicator of well-being because it promotes positive affect, which buffers youth from the negative effects of stressful events (Gilman and Huebner 2006). Accordingly, we use data from the California Healthy Kids Survey (CHKS)^3^ core module for 7th, 9th, and 11th graders because it assesses several elements of life satisfaction, as well as youth access to caring relationships with peers and adults (WestEd 2006; Austin et al. 2010).

The CHKS survey, offers a rich dataset on students’ self-reported experiences, and is the only large-scale California youth survey to do so that aims for representativeness at a geographic scale smaller than a county. However, the dataset also presents some notable limitations.

- The survey is typically not administered at charter schools, alternative or community day schools, home school programs or private schools.^4^ This could bias composite well-being scores in either direction.
- Actual school and district response rates do not always meet the threshold required to obtain valid and representative data (see Appendix 1 for rates by district and grade level).
- Data are missing for some districts, and school district boundary changes complicate accurate geographic representation.^5^
- Preliminary comparison of survey response data and district enrollment data suggests that in many districts white students may be over-represented and students of color under-represented.

All data source limitations should be considered when interpreting the indicator data and using this index.

### Selection of Indicators and Measures

Though there is general consensus on the broad domains of youth well-being, there is much less agreement on which indicators provide the most robust assessment within each domain (Pollard and Lee 2003). Options are often limited by the scarcity of data, as few surveys administered to children assess life satisfaction or other aspects of well-being that cannot be captured through regularly collected assessments, administrative data or demographic data. We draw upon the research literature to help guide our choices of indicators, while also remaining true to our pre-determined criteria of positive indicators and measures (where possible) and smallest possible geographic scale (the school district) in a readily available dataset. Because indices are sensitive to the choice of measures, how they are grouped, and how they are weighted in calculations of summary scores (Land et al. 2001; O’Hare and Bramstedt 2003; Zill 2006), we also conduct principal components analysis to inform indicator development.

We chose indicators associated with the well-being of adolescents across the health, education, social relationships, and community context domains. We analyze these four domains separately, in addition to creating a composite score of well-being, as research suggests that different facets of child well-being make independent contributions to overall status (Zill 2006). Each domain is comprised of one to three indicators, and each indicator includes one or more measures (see Table 1 for the index organization).

**Table 1:** Youth well-being index domains, indicators and measures

Domain	Indicator	Measure	Range	Source
Health	Physical fitness	% youth deemed fit on 6 of 6 physical fitness tests	0–1.0	CDE

	Substance use avoidance	% youth who smoked 0–1 cigarette in their lifetime	0–1.0	CHKS
		% youth who had 0–1 full drinks of alcohol in their lifetime	0–1.0	CHKS
		% youth who used marijuana 0–1 times in their lifetime	0–1.0	CHKS

	Feeling safe	How safe do you feel at school (not very safe to very safe)	1–5	CHKS
		% not bullied at school past 12 months due to race/ethnicity/national origin	0–1.0	CHKS
		% not bullied at school past 12 months due to religion	0–1.0	CHKS
		% not bullied at school past 12 months due to gender	0–1.0	CHKS
		% not bullied at school past 12 months due to sexual orientation	0–1.0	CHKS
		% not bullied at school past 12 months due to physical or mental disability	0–1.0	CHKS
		% not bullied at school past 12 months for other reasons	0–1.0	CHKS

Education	High school graduation	Proportion graduating from high school (as 1 minus 4-year dropout rate)	0–1.0	CDE

	University ready	Proportion high school graduates passing state university prerequisites	0–1.0	CDE

Social relationships	Positive relationships	There is an adult outside school or home whom I trust (not true to very true)	1–4	CHKS
		Teacher/other adult at my school who really cares about me (not true to very true)	1–4	CHKS
		Outside home and school, there is an adult who really cares about me (not true to very true)	1–4	CHKS
		I feel close to people at this school (strongly disagree to strongly agree)	1–5	CHKS
		I am happy to be at this school (strongly disagree to strongly agree)	1–5	CHKS
		I feel like I am part of this school (strongly disagree to strongly agree)	1–5	CHKS

Community context	Material resources	% 12–17 year olds in households with income at least 300% federal poverty line	0–1.0	Census

	Community involvement	Belong to clubs, teams, church or other group activities (not true to very true)	1–4	CHKS
		Involved in music, art, literature, sports, hobbies (not true to very true)	1–4	CHKS
		Help other people (not true to very true)	1–4	CHKS

#### Health Domain

In the health domain, we examine the results of the Physical Fitness Test administered to California public school 9th graders in 2006–2007 (California Department of Education 2008) to assess physical functioning and fitness. This measure is the percentage of youth who were in the “Healthy Fitness Zone” for all six of the following tests: aerobic capacity, abdominal strength and endurance, upper body strength and endurance, body composition, trunk strength, and back and shoulder flexibility. We also use CHKS questions about avoidance of cigarettes, alcohol and drugs to assess healthy choice-making. These three data elements, when combined, create an indicator representing the percentage of youth who have mostly abstained from substance use in their lifetime. The third indicator in the health domain includes school safety and freedom from harassment based on real or perceived aspects of personal/group identity at school. We employ several CHKS questions as measures. One question asks how safe students feel at school; others ask how often they have been bullied due to their race/ethnicity, gender, religion, sexual orientation, disability or for any other reason. Bullying has been linked to poor mental health among adolescents (Rigby 2000) and all questions are associated with subjective well-being assessments (Kerr et al. 2011; Valois et al. 2001).

#### Education Domain

To assess basic educational outcomes, we use data from the California Department of Education on high school graduation rates (California Department of Education 2008). The extent to which youth in our region have the option of pursuing college educations and reaping the social and economic benefits of higher education is assessed based on the proportion of high school graduates who have completed course requirements to attend a 4-year public university in California (California Department of Education 2008).^6^

#### Social Relationships Domain

The social relationships domain consists of a single indicator that measures youth engagement in positive relationships across multiple contexts. Healthy relationships with parents are highly correlated with adolescents’ own sense of their well-being (Fattore et al. 2007; Gilman et al. 2000), but the CHKS core module for secondary school students does not include questions about parental relationships. We build on research demonstrating the importance of other adult relationships as well (Burton and Phipps 2008), analyzing data from three CHKS questions that assess the presence of high quality relationships with adults in and out of school. We also employ CHKS questions that assess school engagement and a sense of belonging (Finn 1993; Wang and Holcombe 2010).

#### Community Context Domain

Our analysis of young people’s community contexts includes two indicators: material resources and community involvement. The material resources indicator is comprised of a single measure assessing the proportion of young people growing up in households with adequate financial resources, while community involvement is assessed using a combination of three measures.

Though youth do not tend to emphasize family socioeconomic status when asked to assess their quality of life (Burton and Phipps 2008), sufficient family income is correlated with educational, health and social well-being amongst youth (Moore et al. 2008). In addition, neighborhood effects research suggests that concentrated economic well-being is a direct predictor of many positive youth outcomes, as well as social processes associated with youth well-being (Leventhal and Brooks-Gunn 2000; Sampson et al. 2002).

To construct a threshold of “adequate” household resources, we draw on cost of living estimates associated with a “living wage” for families with children in California, generated by the “Living Wage Calculator” (for discussion of this tool and its development, see Farrigan and Glassmeier (2003))^7^; across various family configurations these estimates are approximately three times the federal poverty level. Therefore, we include the percentage of youth ages 12 to 17 years living in households earning at least 300% of the federal poverty rate, using data from the U.S. Census Bureau’s five-year American Community Survey estimates for 2005–2009, tabulated at the school district level (U.S. Census Bureau 2010).

We examine community engagement by including three CHKS questions that ask youth whether they are involved in out-of-school extracurricular activities such as clubs, sports, and/or the arts, and whether they help other people in their community. Several studies find that participation in extracurricular activities and voluntary organizations facilitates positive development among youth by providing structured environments where youth associate with peers under the supervision of supportive adults (Eccles and Barber 1999; Fredricks and Eccles 2006). Youth who report more involvement in extracurricular activities also report greater life satisfaction (Gilman 2001; Gilman et al. 2004) and show higher levels of motivation (Larson et al. 2004).

### Index Construction and Data Analysis

#### Data Standardization

A high priority objective of this index was to enable users to compare local youth well-being with an optimal or “absolute” level of well-being, while a secondary interest was to identify places where young people appear to be faring especially well or poorly. While Vandivere and McPhee (2008) suggest that employing an index construction strategy based on z-scores would be most appropriate for comparing well-being across places, this approach does not enable comparison with an absolute measure. In addition, while statistically sound, the z-score approach requires additional explanation to a non-academic audience and precludes side-by-side comparison of maps that may be generated to show the same analysis at another point in time. To address these concerns, we employed the following approach to index construction.

For each of the four domain areas—health, education, social relationships, and community context—we have constructed analyses of the proportion of youth that fall into the most positive category across indicators. The index presents a composite score based on all four domains. Original data elements came in multiple forms, ranging from Likert scales of varying size to percentages.

In order to create compatible data elements, we employed a linear scaling transformation (Booysen 2002) in which each item was converted to a percentage of the best possible score. For example, with a Likert scale ranging from 1 to 3 and an average response of 2.4, the resulting percentage would account for the score minus the lowest possible score (2.4-1) divided by the range of possible scores (3-1) to equal 70%.

#### Data Aggregation

Once the measures were standardized, we used results from correlational and principal components analyses to determine which items assess the same construct, aggregating those items into a single indicator by averaging over them. This avoids the possibility that any one construct will be overrepresented in the final index through the appearance of redundant measures. Measures subject to aggregation are described below.

In the health domain, the substance use questions all loaded onto a single factor, with factor loadings between 0.48 and 0.54. The internal reliability coefficient for the resulting indicator was high (Cronbach’s alpha = 0.85). The freedom from bullying questions, with the exception of bullying due to race, loaded onto a second factor, though with more moderate loadings (0.31–0.43). Freedom from race bullying and school safety loaded onto a third factor, but they also loaded onto the same factor as the other bullying questions, albeit more weakly (0.25–0.26).We chose to combine all of these items into a single indicator of school safety which has good reliability (Cronbach’s alpha = 0.88), and compares favorably to the reliability for an indicator which excludes race-based bullying and school safety (Cronbach’s alpha = 0.85).

The six measures comprising the social relationships domain are all highly interrelated and load strongly onto a single factor. Therefore, we combined them into a single indicator which has a Cronbach’s alpha value of 0.97.

The community involvement indicator was formed from three measures which all load onto a single factor: belonging to clubs, teams, church or other group activities, involvement in music, art, literature, sports or hobbies, and helping other people. The resulting indicator has a reliability coefficient of 0.92. While the measure of household income adequacy also loads onto a factor with the community involvement indicators, we chose to employ this measure in a separate “material resources” indicator because it does not load as strongly and research literature suggests a complex relationship between income and engagement (Ginwright and Cammarota 2007; Yosso 2005).

#### Index Weighting

We then use a stepwise approach in which we equally weight indicators in each domain score, and then the domains are equally weighted in the overall index score. In studies of children’s and youth well-being, it is common to use multiple indicators across each domain of well-being without overweighting any one domain in the absence of compelling theoretical or practical reasons to do so (Hagerty and Land 2007; Zill 2006). Indices using this approach include those created by Bradshaw et al. (2007), Hur and Testerman (2012), Land et al. (2001), and Moore et al. (2007). One recent exception is a well-being index developed in Canada using domain weights based on their subjective relative importance as determined by a panel of experts assembled by the study’s authors (Martin et al. 2011). However, the authors report that scores calculated using equal weights were highly correlated with scores calculated using the subjective weights (Pearson’s *r* = .961, *p* < .01), suggesting that equal weights are an optimal choice when study constraints do not permit more elaborate techniques for determining the relative importance of domains.^8^

#### Index Variability

The aggregate index of youth well-being by school district showed variability in the range of 26%, demonstrating substantial differences in well-being across school district boundaries.

### Mapping

Index data were imported into GIS software (ArcMap v10.0, Esri, Redlands, CA) and joined to a geographic file representing merged Unified School District boundaries and Secondary School District boundaries. While this index employs school districts as the geographic unit of analysis, it is important to note that the index is not an analysis of schools and school districts, but rather youth well-being in the areas bound by these school districts. That said, United States school district boundaries often reflect municipal boundaries or clusters of incorporated and unincorporated areas linked by shared service providers, land use policies, etc. and therefore present a policy-relevant geography with respect to youth well-being.

For each of the four domains and the aggregate index, a map representing the distribution of the data was symbolized. In each map, the data are broken down into 10% intervals that range from a low of 20% to the maximum possible score, 100%. This classification scheme was chosen to permit comparisons across domains, enable interpretation by a variety of non-academic audiences, and facilitate the detection of change over time.

## Results

**Fig. 1:**
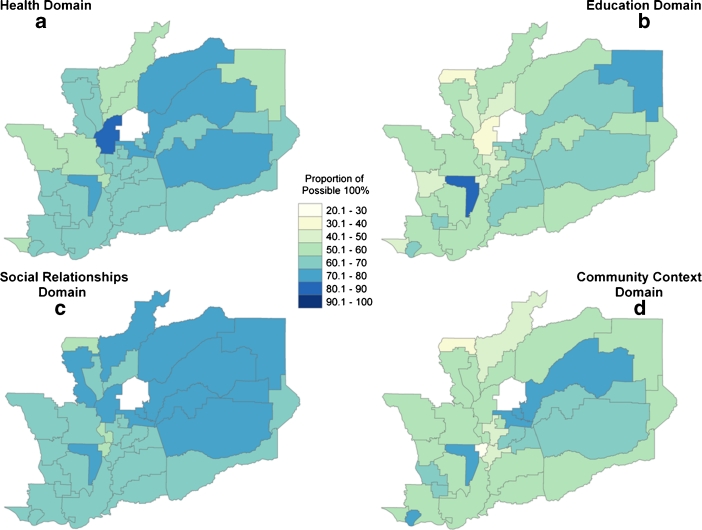
Four domain maps depict individual domain scores for the region’s school districts (the central white area has no score because the school district did not generate CHKS data)

**Fig. 2:**
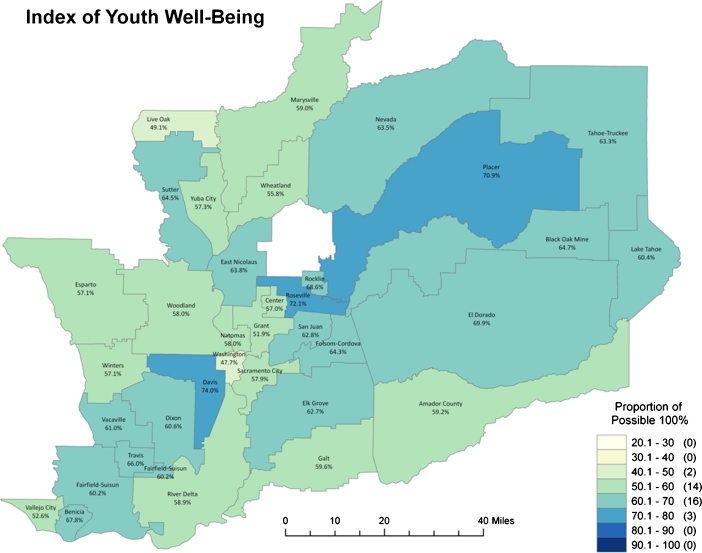
The index of youth well-being provides composite scores for each school district reflecting the four domains: health, education, social relationships and community context

The following maps depict the resulting regional analyses of each domain (Fig. 1) and the cumulative index (Fig. 2).

In the Health Domain (Fig. 1a), the physical fitness indicator ranged widely; 3% to 92% of public school district students met “Healthy Fitness Zone” criteria. The ranges for youth avoidance of marijuana, alcohol and cigarettes were 64–97%, 52–91% and 78–98% respectively. The indicator for feeling safe ranged from 75% to 88%. The district scoring highest in the Health Domain did so primarily due to their students’ higher performance on the physical fitness measure. The majority of the region’s school districts received low overall physical fitness scores, but many areas’ Health Domain scores were elevated by youth reporting feeling safe.

In the Education Domain (Fig. 1b), scores for the indicator describing completion of high school ranged from 49% to 97% while the scores for college-readiness (high school graduates who completed A-G requirements for college entry) ranged from 0% to 66%. Districts with the highest scores showed the highest levels of college readiness. The areas receiving the lowest overall score in this domain did so due to the relatively low numbers across both indicators.

The indicator in the Social Relationships Domain (Fig. 1c) includes access to a trusted adult (range 64%–100%), access to caring teachers (44%–67%) and adults (74%–100%, and a sense of engagement with the school (52%–71%). Low-scoring areas primarily had less access to a trusted adult, and generally scored low in all measures.

The Community Context Domain (Fig. 1d) map presents analysis across the indicators of adequacy of material resources (9% to 79%) and community participation (49% to 76%). Adequacy of financial resources was the primary driver of the overall Community Context Domain scores and was associated with having high scores in community participation (Pearson correlation coefficient 0.66). However, despite the influence of income on the Community Context Domain, we found that overall Community Context Domain scores changed minimally when we calculated them with and without the income measure (Pearson correlation coefficient 0.88). The lowest scores in the Community Context Domain had low scores for both indicators.

The well-being index map (Fig. 2) presents composite scores based on the scores across all four domains. We calculated scores with and without the income measure and found that this variable did not exert excessive influence on the cumulative index (Pearson correlation coefficient 0.98; map without income measure not displayed). Across the areas bound by the 35 unified and secondary school districts in the region, young people in three of them appear to be experiencing well-being at relatively high rates. Youth living in much of the region appear to be experiencing only moderate levels of well-being, with 16 out of 35 school districts scoring in the range of 60.1–70% out of a possible 100%.

## Discussion

Together these maps demonstrate that there are spatial disparities in youth well-being across the 9-county Capital Region that manifest at a sub-county scale. Disparities play out across each domain of well-being, often in similar geographic patterns. Our analysis shows that there is significant room for improvement throughout the region, with no district receiving a score higher than 74% out of a possible 100%. In light of the under-representation of non-traditional students in the CHKS dataset (including incarcerated youth, school-age youth who left without graduating high school, young people attending alternative/community day schools), these maps likely present an optimistic snapshot.

For the purpose of this article, we focus our discussion on the limitations and utility of this index, as opposed to the specific patterns that arise in California’s Capital Region. We acknowledge that the index omits certain key indicators that we would have liked to incorporate, but for which data were unavailable, or unavailable at an appropriate scale. For example, while we consider youth connectedness to caring and supportive adults in the school and community, we do not include robust measures of relationships with parents, peers, or partners, which numerous studies find are pivotal factors in youth well-being (Gilman 2001; Greenberg et al. 1983; Youngblade et al. 2007; Burton and Phipps 2008). We were also unable to include information about positive gender, ethnic and sexual identity development (Swanson et al. 2002). Our indicator data for the education domain focuses heavily on school, and not at all on development of other critical life skills and knowledge (e.g. knowing how to navigate systems, manage personal finances, etc.). In addition, this analysis is constrained by the paucity of data on positive indicators of youth well-being (as opposed to measures of negative behaviors and outcomes) that have been noted by many others (Lippman et al. 2009; Moore et al. 2008; Morrow and Mayall 2010).

The utility of this index at broader scale is limited by its dependence on a data source particular to California (CHKS). The reliability of the index would be enhanced by improving administration of CHKS through increasing response rates and ensuring alternative school inclusion and representation of “non-traditional” students.

As with all indices of this type, assessing validity is a challenge. The selection of index measures was driven by research, but as noted above our choices were limited by the availability of data at the appropriate scale; it is possible that some domains of well-being are not adequately represented in the index. Indices are sensitive to the items included, how they are grouped, and how they are weighted in calculations of summary scores (O’Hare and Bramstedt 2003; Zill 2006; Land et al. 2001). We realize that our ‘stepwise equal weighting’ method ultimately created unequal weights for our indicators within domains due to differing numbers of indicators within a domain (from one to three). Our primary goal was to weight the domains equally to be consistent with prior research and to ensure that the indicators we included provided the most complete picture possible of well-being for each domain. Our use of principal components analysis helped to ensure that indicators were grouped properly without significant redundancy in the effects they measured.

As with any index created by summing multiple items into a composite measure, our index of well-being is subject to the problem of compensatability. A high score in one domain may offset a low score in another domain, leading to a moderate overall score for a school district. Another school district may achieve the same overall score through the combination of moderate sub-domain scores. These two districts, though qualitatively different, would appear similar given their index scores. Similarly, two districts could achieve the same domain score through different combinations of values on the component indicators. To offset this shortcoming, we provide information about the distribution of domain scores, and within domains, indicator scores. However, the compensatability problem limits the ability of the index to convey detailed information about how school districts are faring in the absence of this additional information.

An analytic limitation in this research is our inability to test statistical significance between well-being scores by school districts. Since we collect the data at the school district level (thus one observation per variable per school district), at a single point in time, we are unable to assess the stability of our estimates or the error in each observation. This is a consequence of our choice to use the smallest possible geographic area as the analytic enumeration unit in order to provide a higher level of relevance to policy-makers and accommodate annual trend analysis.

The school district scale may still mask important geographic disparities, a consequence of the modifiable areal unit problem (MAUP). The MAUP is a source of statistical bias in aggregated data, caused by the choice of boundary used in the analysis (Openshaw 1984). In this case, the data are originally compiled at a more detailed level, the school, although CHKS administration is designed to be representative of the school district. We chose school districts since they represent clear geographic boundaries, they exist at sub-county scale, and they are areas within which policy decisions are made.

It is difficult to assess the validity of a multidimensional index because it is not clear what criteria should be used to judge it (see Zill 2006) for more on the ability of longitudinal studies to assess well-being indices). However, a set of Capital Region-focused qualitative studies of youth well-being (*n*(youth) = 16, *n*(adult allies) = 59) offered one external basis for assessment, and patterns captured in this index do reflect local and regional descriptions that emerged through that research (Benner et al. 2010; Erbstein et al. 2010; Geraghty 2010; London et al. 2011; Owens et al. 2010). Going forward, it will be important to compare this index to others, to assess its ability to predict various concurrent and future outcomes, and to assess its utility as a tool for policy-makers, youth-focused practitioners and youth advocates.

Despite the limitations discussed, the index captures key aspects of youth well-being, including factors that are known to predict successful and healthy transitions to adulthood, as well as factors that youth themselves consider important. An index based on all of these indicators provides a snapshot of the state of youth in our region and as such holds potential as a tool for promoting regional learning and action. Our initial translational work suggests that the maps provide a powerful basis for discussion amongst local and regional institutions that are relevant to youth well-being. Presenting complex data in a relatively straightforward visual format enables a wide range of stakeholders to understand and engage with the findings. The comprehensive orientation to well-being embodied by the index offers a basis for the types of cross-sector discussion and coordination that are increasingly viewed as necessary to create the conditions that foster healthy adolescence. Identifying settings where youth appear to demonstrate more positive outcomes—both cumulatively as well as within domains—provides a starting point for identifying and sharing contributing policies and practices. Areas of relative strength and weakness within localities can be examined by considering domain-specific scores. Finally, the availability of regularly updated data for these indicators will enable ongoing monitoring of youth well-being over time.

By offering geographically-specific information about the status of youth, strengths and challenges, potential areas for investigation and investment, and emerging spatial disparities, the index also offers a basis for targeted action by policy-makers, program leaders and advocacy groups. The relatively local scale of the data and resulting analyses lend themselves to facilitating work at the municipal, county, and regional scales, as well as collaboration across them.

## Conclusion

This index documents geographic disparities in the overall well-being of young people, as well as variation within the domains of Health, Education, Social Relationships and Community Contexts. While not without limitations, the index holds potential to motivate and facilitate local and regional action on behalf of youth. To facilitate local and regional index utility we pursue several strategies, including adopting a positive orientation to well-being, integrating subjective and objective measures, employing a sub-county geographic scale of analysis and display, and designing the maps and indices to facilitate their accessibility and use over time.

## Appendix 1. The California Healthy Kids Survey (CHKS)

In California, Local Education Agencies (LEAs) and County Offices of Education (COEs) that accept funds under the federal Title IV Safe and Drug Free Schools and Communities program or the state Tobacco Use Prevention Education (TUPE) program must assess the conditions and consequences of risk factors such as violence and drug use in the elementary and secondary schools served. The California Department of Education partnered with WestEd, a national education laboratory, to develop CHKS, a survey that meets these requirements. WestEd assists schools in fielding the survey, analyzing the results, and maintaining the data, which they also make available to researchers with appropriate restrictions to protect student confidentiality. Participating agencies are expected to conduct a representative district-level survey every 2 years with students in grades 5, 7, 9, and 11. This includes charter schools that receive Title IV funding through the district, as well as continuation and community day schools students as determined by the CHKS sampling plan.

The survey itself consists of a grade-specific core module and optional supplemental modules. Because not all participating school districts administer the supplemental modules, we included only questions from the core module. Given our focus on adolescents, we employed 7th, 9th and 11th grade data. The middle and high school core modules contain similar questions, although the high school module asks more detailed questions about substance use, for example. Where necessary, we collapsed questions or response categories in the high school module to correspond to similar questions and response categories in the middle school module.

Data were analyzed for high school and unified school districts. In cases where CHKS was administered in elementary school districts that contain middle schools, or charter schools that serve middle and high school youth, we aggregated data from these feeder districts to their associated high school district. Table 2 displays the involved elementary school districts and the high school districts with which their data were linked.

**Table 2:** Participating districts and feeder district aggregation patterns

District name	Linked high school district
Buckeye Union Elementary	El Dorado Union High
Camino Union Elementary	El Dorado Union High
Gold Oak Union Elementary	El Dorado Union High
Gold Trail Union Elementary	El Dorado Union High
Latrobe	El Dorado Union High
Mother Lode Union Elementary	El Dorado Union High
Pioneer Union Elementary	El Dorado Union High
Placerville Union Elementary	El Dorado Union High
Pollock Pines Elementary	El Dorado Union High
Rescue Union Elementary	El Dorado Union High
Chicago Park Elementary	Nevada Joint Union High
Clear Creek Elementary	Nevada Joint Union High
Grass Valley Elementary	Nevada Joint Union High
Nevada City Elementary	Nevada Joint Union High
Pleasant Ridge Union Elementary	Nevada Joint Union High
Pleasant Valley Elementary	Nevada Joint Union High
Union Hill Elementary	Nevada Joint Union High
Twin Ridges Elementary	Nevada Joint Union High
Ready Springs Union Elementary	Nevada Joint Union High
Alta-Dutch Flat Union Elementary	Placer Union High
Auburn Union Elementary	Placer Union High
Colfax Elementary	Placer Union High
Foresthill Union Elementary	Placer Union High
Loomis Union Elementary	Placer Union High
Newcastle Elementary	Placer Union High
Dry Creek Joint Elementary	Roseville Joint Union High
Eureka Union	Roseville Joint Union High
Roseville City Elementary	Roseville Joint Union High
Arcohe Union Elementary	Galt Joint Union High
Galt Joint Union Elementary	Galt Joint Union High
Elverta Joint Elementary	Grant Joint Union High
Twin Rivers Unified	Grant Joint Union High
Browns Elementary	East Nicolaus Joint Union High
Marcum-Illinois Union Elementary	East Nicolaus Joint Union High
Pleasant Grove Joint Union	East Nicolaus Joint Union High
Brittan Elementary	Sutter Union High
Franklin Elementary	Sutter Union High
Meridian Elementary	Sutter Union High
Camptonville Elementary	Nevada Joint Union High
Wheatland	Wheatland Union High
Plumas Lake Elementary	Wheatland Union High

WestEd generated response rates by district and grade level, and for non-traditional (NT) students for Healthy Youth/Healthy Regions in August 2010 (see Table 3 below). WestEd strongly recommends an overall 70% threshold to obtain valid and representative data at the district scale (http://chks.wested.org/about/faq_fees#enough, guidelines downloaded September 20, 2010).

**Table 3:** 2006–2008 Capital Region CHKS response rates (by grade level and for non-traditional students)

District	7th grade	9th grade	11th grade	NT students
Amador Co.				
Amador County Unified	90.07%	77.02%	55.59%	27.27%
El Dorado Co.				
Buckeye Union Elementary	88.10%	–	–	–
Camino Union Elementary	–	–	–	–
El Dorado Union High	57.14%	–	59.69%	85.14%
Gold Oak Union Elementary	72%	–	–	–
Gold Trail Union Elementary	87.95%	86.01%	–	–
Indian Diggings Elementary	–	–	–	–
Lake Tahoe Unified	57.58%	–	74.84%	62.16%
Latrobe	76.69%	–	–	–
Mother Lode Union Elementary	62.71%	–	–	–
Pioneer Union Elementary	95.50%	–	–	–
Placerville Union Elementary	84.09%	–	–	–
Pollock Pines Elementary	87.94%	–	–	–
Rescue Union Elementary	81.82%	–	–	–
Silver Fork Elementary	–	–	–	–
Black Oak Mine Unified	72.28%	–	76.28%	26.25%
Nevada Co.				
Chicago Park Elementary	43.75%	–	–	–
Clear Creek Elementary	81.07%	–	–	–
Grass Valley Elementary	85.96%	–	–	100.00%
Nevada City Elementary	35.14%	45.95%	–	46.15%
Nevada Joint Union High	77.18%	–	80.66%	56.85%
Pleasant Ridge Union Elementary	67.03%	–	–	0.00%
Pleasant Valley Elementary	86.96%	66.67%	–	–
Ready Springs Union Elementary	27.27%	–	33.33%	–
Union Hill Elementary	91.07%	–	–	–
Twin Ridges Elementary	70.53%	–	–	–
Placer Co.				
Ackerman Elementary	46.15%	–	–	–
Alta-Dutch Flat Union Elementary	75.73%	–	–	–
Auburn Union Elementary	66.04%	–	–	–
Colfax Elementary	78.04%	–	–	–
Dry Creek Joint Elementary	85.10%	–	–	–
Eureka Union	0.00%	–	–	–
Foresthill Union Elementary	86.73%	–	–	–
Loomis Union Elementary	64.81%	–	–	–
Newcastle Elementary	–	–	–	–
Ophir Elementary	–	–	–	–
Placer Hills Union Elementary	–	84.15%	–	–
Placer Union High	86.27%	79.09%	73.47%	33.33%
Roseville City Elementary	–	68.57%	–	–
Roseville Joint Union High	88.52%	79.57%	69.32%	52.16%
Tahoe-Truckee Joint Union	65.78%	61.63%	73.44%	29.17%
Western Placer Unified	60.42%	–	64.20%	0.00%
Rocklin Unified	58.99%	–	76.59%	82.86%
Sacramento Co.				
Arcohe Union Elementary	89.28%	84.01%	–	–
Elk Grove Unified	82.05%	–	71.87%	28.68%
Elverta Joint Elementary	84.32%	82.29%	–	–
Folsom-Cordova Unified	60.05%	–	73.25%	79.36%
Galt Joint Union Elementary	–	61.22%	–	–
Galt Joint Union High	77.74%	74.74%	67.90%	86.25%
Grant Joint Union High	–	–	–	–
North Sacramento Elementary	–	–	–	–
Rio Linda Union Elementary	–	–	–	–
River Delta Joint Unified	–	–	64.08%	12.24%
Sacramento City Unified	77.85%	67.25%	67.32%	57.92%
San Juan Unified	69.73%	72.14%	60.10%	59.33%
Center Joint Unified	60.34%	63.18%	82.47%	66.96%
Natomas Unified	84.44%	80.49%	63.83%	62.12%
Twin Rivers Unified	58.95%	71.43%	59.82%	68.67%
Solano Co.				
Benicia Unified	98.02%	95.49%	68.72%	40.30%
Dixon Unified	90.73%	60.79%	99.17%	–
Fairfield-Suisun Unified	0.00%	0.00%	75.77%	71.39%
Travis Unified	85.53%	84.76%	87.73%	78.33%
Vacaville Unified	83.82%	55.59%	91.38%	100.00%
Vallejo City Unified	74.14%	–	56.29%	68.15%
Sutter Co.				
Brittan Elementary	90.91%	–	–	–
Browns Elementary	–	76.14%	–	–
East Nicolaus Joint Union High	76.36%	–	71.43%	–
Franklin Elementary	70.83%	55.48%	–	–
Live Oak Unified	85.00%	–	66.90%	52.94%
Marcum-Illinois Union Elementary	92.31%	–	–	–
Meridian Elementary	95.65%	–	–	–
Nuestro Elementary	–	–	–	–
Pleasant Grove Joint Union	–	–	–	–
Sutter Union High	28.57%	–	60.20%	–
Winship-Robbins	81.56%	69.15%	–	–
Yuba City Unified	85.82%	78.01%	64.52%	17.81%
Yolo Co.				
Davis Joint Unified	70.13%	88.89%	82.54%	68.30%
Esparto Unified	63.62%	45.40%	86.89%	67.86%
Washington Unified	85.83%	83.55%	60.74%	24.81%
Winters Joint Unified	72.67%	55.39%	81.45%	35.71%
Woodland Joint Unified	–	–	57.30%	65.91%
Yuba Co.				
Camptonville Elementary	–	–	–	–
Marysville Joint Unified		81.25%	21.43%	–
Plumas Lake Elementary	89.17%	70.83%	75.73%	–
Wheatland	–	–	74.19%	26.00%
Wheatland Union High	51.01%	–	–	42.16%

## Footnotes

1 Healthy Youth/Healthy Regions was a study commissioned and funded by Sierra Health Foundation with additional funding from The California Endowment. For more information on the Capital Region and Healthy Youth/Healthy Regions, see http://regionalchange.ucdavis.edu/projects/healthy-youth-healthy-regions.

2 We searched the literature for other indices of child well-being using keyword searches in the Social Science Citation Index and snowball methods. Keywords employed were (child OR youth OR adolesc*) AND (index or indicator) AND (well-being).

3 CHKS is similar to the United States’ Youth Risk Behavior Surveillance System national survey, but includes an emphasis on both risk behavior and protective factors. It is administered through a partnership between CDE and WestEd, a national education lab, in 5th, 7th, 9th, and 11th grade at all public schools receiving funds under the federal Title IV Safe and Drug Free Schools and Communities program of NCLB or the state Tobacco Use Prevention Education (TUPE) program. For additional information on CHKS, see Appendix 1.

4 In 75% of districts serving non-traditional students, this population’s response rate was lower than that of the general student population, in many cases by as much as 40 to 50 percentage points.

5 In the Capital Region, CHKS data were unavailable for one district. In the case of one new district that resulted from recent consolidation of two smaller districts, the original district boundaries were employed in data analysis and mapping.

6 The data for two school districts, Natomas Unified and Center Joint Unified, showed that zero graduates had completed college requirements in 2006–07, but this appears to be a reporting anomaly, as one district reported that significant numbers of 2007–08 graduates had completed the college requirements, and both reported having many college-ready graduates in the 2005–06 school year. We opted to use data from 2005 to 2006 for these two districts to overcome this anomaly, for which we could find no explanation on the CDE website that provides access to these data (California Department of Education 2007).

7 Downloaded from http://www.livingwage.geog.psu.edu/states/06 on March 23, 2011. Estimates are based on the latest data available as of November 2008.

8 Hagerty and Land (2007) demonstrate that when there are strong positive correlations between items in an index, overall index scores tend to be correlated across observations independent of the weighting scheme. This phenomenon may explain why equal weighting and subjective weighting techniques produced such similar results in the study by Martin et al. (2011). The choice of weights is more consequential when there are many negative item correlations, depending on the distribution of weights themselves (Hagerty and Land 2007).
